# Impact Dynamics of Non-Newtonian Droplets on Superhydrophobic Surfaces

**DOI:** 10.1021/acs.langmuir.3c00043

**Published:** 2023-04-11

**Authors:** Mehdi H. Biroun, Luke Haworth, Hossein Abdolnezhad, Arash Khosravi, Prashant Agrawal, Glen McHale, Hamdi Torun, Ciro Semprebon, Masoud Jabbari, Yong-Qing Fu

**Affiliations:** † Department of Chemical Engineering, University College London, London WC1E 7JE, U.K.; ‡ Faculty of Engineering and Environment, University of Northumbria, Newcastle upon Tyne NE1 8ST, U.K.; § School of Mechanical Engineering, Iran University of Science and Technology, Tehran 13114-16846, Iran; ∥ Institute for Multiscale Thermofluids, School of Engineering, University of Edinburgh, Kings Building, Edinburgh EH9 3FB, U.K.; ⊥ School of Mechanical Engineering, University of Leeds, Leeds LS2 9JT, U.K.

## Abstract

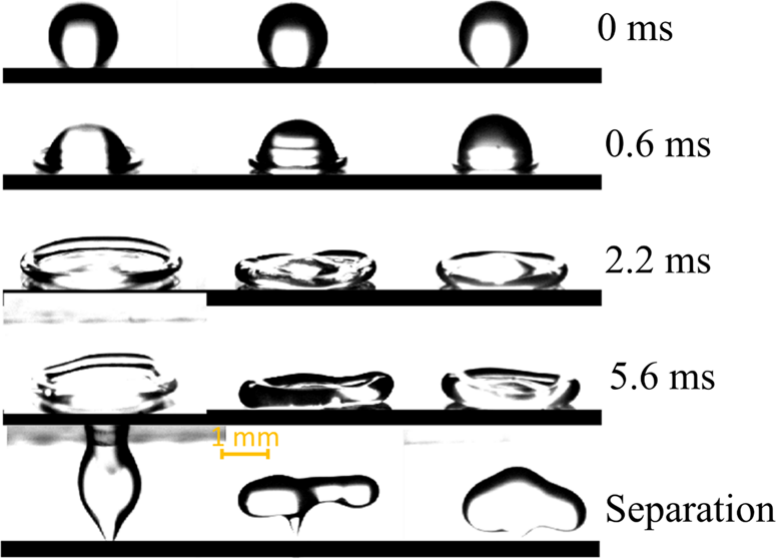

Droplet impact behavior on a solid surface is critical for many industrial applications such as spray coating, food production, printing, and agriculture. For all of these applications, a common challenge is to modify and control the impact regime and contact time of the droplets. This challenge becomes more critical for non-Newtonian liquids with complex rheology. In this research, we explored the impact dynamics of non-Newtonian liquids (by adding different concentrations of Xanthan into water) on superhydrophobic surfaces. Our experimental results show that by increasing the Xanthan concentration in water, the shapes of the bouncing droplet are dramatically altered, e.g., its shape at the separation moment is changed from a conventional vertical jetting into a “mushroom”-like one. As a result, the contact time of the non-Newtonian droplet could be reduced by up to ∼50%. We compare the impact scenarios of Xanthan liquids with those of glycerol solutions having a similar apparent viscosity, and results show that the differences in the elongation viscosity induce different impact dynamics of the droplets. Finally, we show that by increasing the Weber number for all of the liquids, the contact time is reduced, and the maximum spreading radius is increased.

## Introduction

Since the pioneering work of Worthington,^1^ droplet impact on solid surfaces has attracted extensive attention, as it commonly occurs in nature and is critical for various industrial applications such as food production, agriculture, spray coating, lab-on-a-chip, and three-dimensional (3D) printing.^2−5^ In many industrial applications such as self-cleaning,^6^ anti-icing,^7^ and antierosion,^8^ various types of water-repellent surfaces with minimum droplet impact contact time have been developed. Recently, studies have been focused on innovative methods for modifying solid surfaces to reduce contact time. Different types of microstructured or nanostructured surfaces, including superhydrophobic surfaces,^7,9^ surfaces with various types of nanobarriers,^10^ textured surfaces with various macrostructures,^11,12^ and convex and concave curved surfaces,^13^ have been used to alter the droplet impact dynamics on the solid surfaces. In addition, researchers have utilized active techniques such as surface acoustic waves^14^ and horizontal substrate motion^15^ to break the symmetry of the droplet during the impact and reduce the contact time. Apart from the contact time reduction, some studies have been focused on surface modifications/designs to alter the impact regimes,^16^ for example, modifying surfaces to achieve superhydrophobicity and avoid droplet deposition on the surface.^17−19^ In contrast, other studies have been focused on preventing droplets from rebounding from the surface.^20^

Recently, non-Newtonian droplet impact on solid surfaces has received significant attention due to their diverse industrial and social applications such as lab-on-a-chip (LOC),^21^ criminology,^22^ coating and spraying,^23^ and agriculture.^20^ However, understanding the physics of the impact of these non-Newtonian liquids and predicting their impact dynamics on different types of surfaces has significant challenges since various types of non-Newtonian liquids show dramatically different and complex rheological behaviors.^24^ Shear-thinning fluids, also known as pseudo-plastics, are one of the most common non-Newtonian liquids and are ubiquitous in industrial and biological processes.^25^ Common examples include ketchup, paints, and blood. The viscosity of shear-thinning liquids is decreased as the shear rate increases. In Newtonian fluids, however, fluid viscosity is independent of the shear rate.^26^ In the viscosity curve of a shear-thinning fluid, there are two Newtonian plateaus: maximum viscosity, where the shear rate is very low (zero-shear viscosity, μ_0_), and minimum viscosity, where the shear rate is very high (infinite-shear viscosity, μ_∞_).^26^ When the shear rate is increased between these upper and lower limits, the viscosity decreases.

Xanthan solutions are shear-thinning liquids with extensive applications in the food industry. A few studies have investigated the impact of the Xanthan solution on a solid surface. For instance, An and Lee^26^ investigated the impact dynamics of Xanthan/water droplets on hydrophilic and hydrophobic surfaces. They observed that the maximum spreading radius and receding velocity for these Xanthan droplets are ∼100% higher than those of the glycerin droplets. They explained that, as a shear-thinning drop spreads, the viscosity decreases from zero-shear viscosity to infinite-shear viscosity and then returns almost to zero shear at its maximum spreading. Under identical impact conditions, the maximum spreading diameter of a shear-thinning drop appears larger than that of a Newtonian drop with identical zero-shear viscosity but smaller than that of a Newtonian drop with identical infinite-shear viscosity. Moon et al.^27^ also studied the spreading and receding characteristics of Xanthan droplets impinging on solid surfaces at different Weber numbers. They noticed that due to the shear-thinning effect and the large zero-shear viscosity, the receding phase of non-Newtonian droplets was much slower.

While some theories have been proposed, none of them have been able to predict the interactions between Xanthan solutions and solid surfaces fully. Thus, more studies are needed to understand the complex behavior of Xanthan solutions. In this study, we investigate the impact of aqueous Xanthan solutions on hydrophobic silica nanoparticle-coated glass surfaces, which exhibit a superhydrophobic behavior. As few studies have focused on spreading and recoiling Xanthan solution droplets on surfaces, our research can provide a detailed explanation of the complex behavior of the non-Newtonian liquid during its impact on hydrophobic surfaces. By doing so, we aim to contribute to developing a predictive theory for the Xanthan solution rheology. The post-impingement behaviors of the droplets with the Newtonian and non-Newtonian droplets were visualized/compared, and the effects of the liquid viscosity and complex liquid rheology were investigated systematically. Our findings suggest that increasing the concentration of Xanthan in water can alter the physical properties of the liquid, leading to a reduction in the droplet contact time on the surface. Xanthan is a natural thickening polymer that can increase the apparent viscosity of the liquid and cause the fluid to behave elastically (especially at high Xanthan concentrations), which can affect the droplet behavior during its impingement. Our results show that up to a particular concentration, increasing the amount of Xanthan in the liquid leads to a shorter contact time between the droplet and the solid surface.

## Experimental Methods

### Non-Newtonian Liquid Preparation

An aqueous Xanthan solution was prepared by diluting Xanthan powder (Sigma-Aldrich 43708-50G) with deionized water (resistivity 18.2 MΩ), following a method reported in ref (28). To prepare the solution, specific amounts of the Xanthan powders were weighed, i.e., 32, 64, 120, 200, 320, and 480 mg, using a Denver Instrument TP-214 scale (resolution: 10^–5^ g). Then, they were added to deionized water to prepare 400, 800, 1500, 2500, 4000, and 6000 ppm solutions. The mixtures were ultrasonically stirred with a magnetic stirrer at 300 rpm for 24 h. One glycerol solution with a 50% volume fraction was prepared by diluting molecular biology-grade glycerol (Sigma-Aldrich) with DI water. In our experiments, we utilized a pure glycerol solution due to its viscosity being comparable to Xanthan solutions with a high concentration of the Xanthan polymer.

### Superhydrophobic Surface Fabrication and Characterization

**Figure 1:**
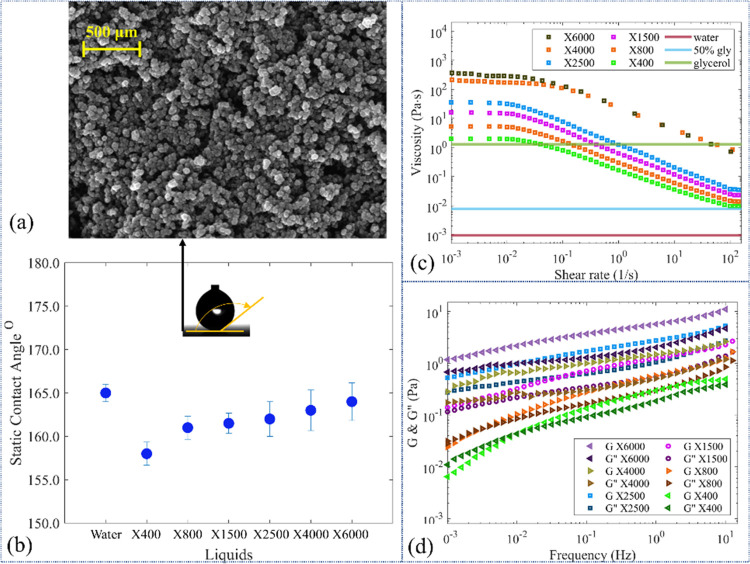
(a) SEM image of the surface of the Glaco-coated surface. (b) Static contact angles of the different Xanthan solution droplets on the Glaco-coated surface. (c) Viscosity of all liquids used in this study as a function of the shear rate and (d) elastic modulus (*G*′) and viscous modulus (*G*″) as functions of the frequency of the Xanthan solutions.

Glass slides were chemically functionalized with silica nanoparticles suspended in isopropanol (IPA) (Glaco Mirror Coat “Zero” from Soft99 Co) to render the surface superhydrophobic. The coating process used was previously reported in ref (29). The process involved spraying the particles five times after allowing IPA to evaporate, leaving a porous layer of ∼2 μm thickness.^30^ A scanning electron microscopy (SEM) image of the Glaco-coated surface is presented in Figure 1a. Figure S1 illustrates two SEM images of the Glaco-coated surfaces before and after undergoing a series of impacts. The images show no significant changes in the structure of the surface. It is worth noting that the roughness of the coatings was measured to be 0.07 μm in previous studies using the same type of coating.^31^

The contact angles of all of the solutions on the superhydrophobic surface were characterized using a drop shape analyzer (Krüss DSA 30). Measurements of advancing and receding contact angles and contact angle hysteresis, Δθ (i.e., the difference between advancing and receding contact angles), were performed using droplets of different solutions with a fixed volume of 3.6 μL. The obtained static contact angles of the droplets for different liquid solutions are shown in Figure 1b. To measure the contact angle hysteresis, a droplet of 4 μL was first placed on the surface. It was then inflated by an additional 2 μL and left to settle for 10 s before being deflated by the same volume. The advancing angle and receding angle, θ_adv_ and θ_rec_, were extracted from the inflation and deflation steps in the procedure. Here, the values of θ_adv_ and θ_rec_ were considered as the largest and smallest possible angle achievable before contact line motion. The obtained values of both dynamic and static contact angles for the various solutions on different surfaces are presented in Table 1.

**Table 1:** Key Parameters of the Liquids in This Study

liquid	advancing contact angle θ_adv_ (degree)	receding contact angle θ_rec_ (degree)	contact angle hysteresis Δθ (degree)	density (kg/m^3^)	surface tension^28,32^ (N/m)
DI water	166.9 ± 3.0	166.0 ± 3.5	0.9 ± 3.3	997.0	0.0707
X400	160.6 ± 4.9	155.4 ± 5.1	5.2 ± 5	998.0	0.0707^28,32^
X800	162.5 ± 4.9	156.5 ± 5.4	6.0 ± 5.1	998.0	0.0707^28,32^
X1500	163.0 ± 4.8	157.6 ± 4.3	5.4 ± 4.6	998.0	0.072^28,32^
X2500	164.8 ± 7.5	158.4 ± 7.2	6.4 ± 7.4	998.0	0.072^28,32^
X4000	165.2± 6.5	158.8 ± 7.0	6.4 ± 6.8	998.0	0.072^28,32^
X6000	165.8± 6.9	159.4 ± 7.2	6.4 ± 7.1	998.0	0.072^28,32^
50% glycerol	168.6	167.2	1.4 ± 1.5	1140.9	0.065^33^
glycerol	164.1	158.9	5.2 ± 0.5	1260.0	0.0634^33^

### Rheology Measurements

A rotational rheometer (Kinexus Lab) equipped with a cone-and-plate geometry (1°/50 mm) was used to determine the rheological properties of the solutions at 20 ± 0.01 °C. The viscosity data of all liquids used in this research as a function of the shear rate are illustrated in Figure 1c. Their elastic modulus (*G*′) and viscous modulus (*G*″) as functions of the frequency of the Xanthan solutions are shown in Figure 1d.

### Droplet Impact Experiments

Droplets of liquids with a volume of 3.6 μL were generated (using an ExiGo 1904-0003 syringe pump) from a hypodermic needle (BD Microlance 19G, with an inner diameter *D*_n_ of 1.5 × 10^–3^ m) mounted on a two-dimensional (2D) positioner. The droplets were released from six different heights, i.e., *H* = 0.05, 0.1, 0.15, 0.2, 0.3, and 0.4 m, with an initial velocity of zero to reach the desired velocity before they were impacted onto the substrate surface, which was placed horizontally. The impact sequences and outcomes were captured from a side view using a high-speed camera (HotShot 1280 CC) with a Navitar 6.0× zoom lens and 0.5× objective lens at 5000 frames per second and a resolution of 432 pixels × 244 pixels. A MATLAB image processing toolbox was used to analyze the temporal evolution of the droplet contact width. The impact tests were carried out under atmospheric conditions (with an ambient temperature of 23 ± 0.5 °C and 30 ± 1% relative humidity). To ensure repeatability, the average value was obtained by repeating each test of the given liquid droplet five times.

## Results and Discussion

### Droplet Impact Results

A set of droplet impact experiments with various liquids were first carried out to investigate the effect of liquid rheology on the droplet impact dynamics. We started our experiments with water droplets and then continued with Xanthan and glycerol solutions. In these experiments, a droplet with a volume of 3.6 μL impacts the solid surface with impact velocities of 1, 1.4, 1.7, 2, 2.4, and 2.8 m/s.

**Figure 2:**
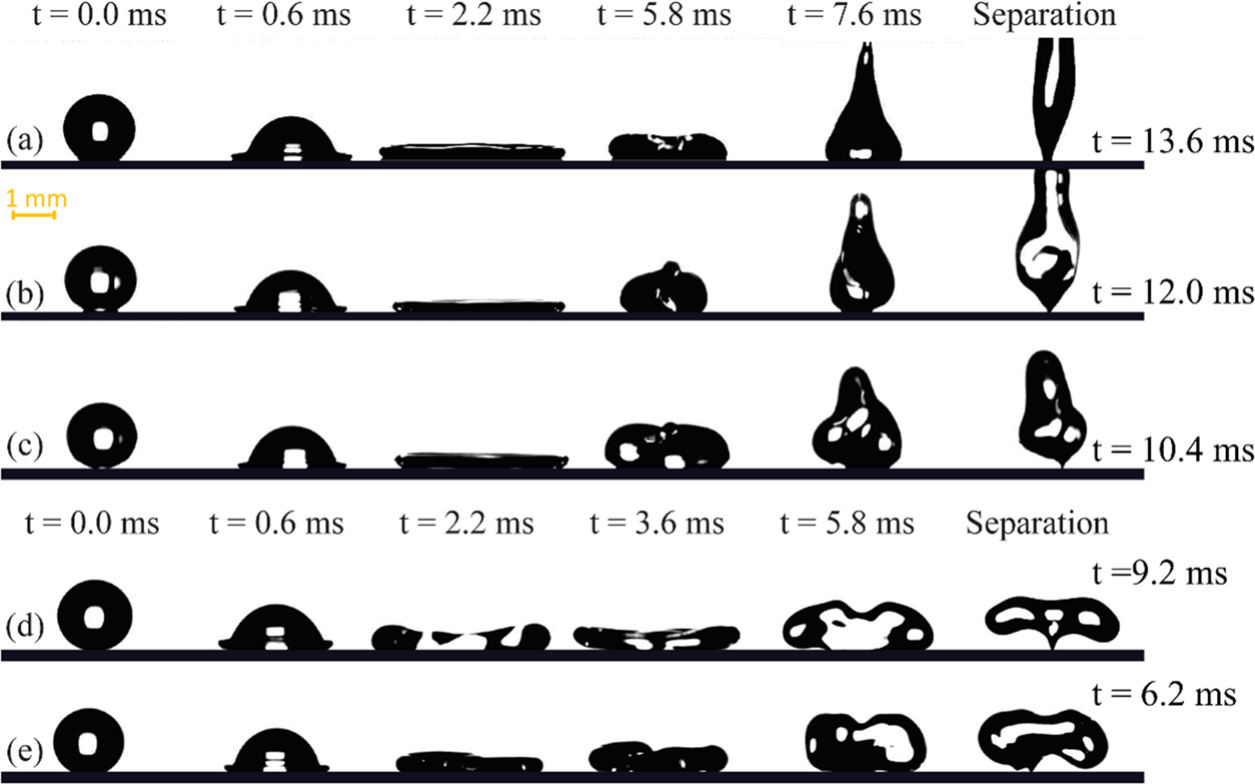
Snapshots of (a) water, (b) X400 solution, (c) X800 solution, (d) X1500 solution, and (e) X2500 solution droplet impact on the Glaco-coated surface. In all experiments, a droplet with a volume of 3.6 μL impacts the solid surface with a velocity of 1.4 m/s.

The first set of analyses examines the effect of Xanthan polymers on the impact dynamics. Figure 2 presents the snapshots of the droplet impingements on the solid surface for both water and Xanthan solution droplets. In the cases shown in Figure 2, the impact velocity of all of the droplets is 1.4 m/s. As expected, the outcome of the water droplet impact was jetting. After the onset of impact, the droplet spreads to a maximum spreading diameter. Then, the rimes of the droplet start to retract toward the impact point until the droplet is separated from the surface as a liquid beam. As the focus of this paper is not to examine the Newtonian droplets impacting onto superhydrophobic surfaces, we recommend that readers refer to the comprehensive analysis conducted by Qu et al.^34^ and Abolghasemibizaki et al.^12^ for a more detailed understanding of these behaviors. By adding Xanthan powder to water, the polymer chains started to show their effect on the impact dynamics (see Figure 2b). In all of the cases, the shape of the droplet was altered during the different stages. At lower Xanthan concentrations, X400 and X800 droplets (see Table 1) did not form a liquid beam at the separation moment, and they were separated from the surface with a pear-like shape. Besides, during the retraction phase, the shape of the droplet became asymmetrical. Dissimilar impact results emerged when the Xanthan concentration was increased to 1500 and 2500 ppm. Figure 2d,e shows that the impact dynamics of liquids were changed significantly by increasing the Xanthan concentration in the solution. Interestingly, these two liquids (i.e., X1500 and X2500 see Table 1) formed a mushroom cap-like shape at the separation moment. From now on, we will refer to this impact regime as the mushroom impact regime.

To provide more detailed information on the impingement process, we conducted additional experiments where the camera was positioned at a 20° angle relative to the horizontal direction. The resulting observations are presented in Figure S2 of the Supporting Information. Our experimental findings showed that the droplet did not break in all of the impact scenarios. In other words, we did not observe any droplet breaking or a subunit separation under the conditions we examined.

At this stage, we were unsure about the effect of the non-Newtonian rheology of the Xanthan solution on the irregular outcome of the impact. Therefore, we further conducted rheology measurement experiments to measure different aspects of the viscosity of the solutions (see Table 2). Generally, the viscosity of the Xanthan solutions increases as the concentration increases. Also, viscosity decreases with the increasing shear rate for these liquids due to their shear-thinning behaviors. A shear-thinning liquid exhibits Newtonian behavior at both low and high shear rates (i.e., the plot of shear stress vs shear rate becomes a straight line) and, on a linear scale, will pass through the origin at low shear rates. Hence, zero-shear viscosity, μ_0_, and infinite-shear viscosity, μ_∞_, refer to the mean apparent viscosities of the fluid at very low and high shear rates, respectively. As a result, the apparent viscosity of a shear-thinning fluid decreases from μ_0_ to μ_∞_ as the shear rate increases (see Figure 1c). Over a limited range of shear rate (or stress), a linear correlation represents the relationship between shear stress and shear rate (plotted on double logarithmic coordinates). The following expression applies to this part of the flow curve:
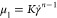
1where μ_l_ is the apparent viscosity at a given shear rate γ̇, *K* is the consistency index, and *n* is the flow behavior index. The smaller the value of *n*, the greater the degree of shear-thinning. Note that when *n* = 1, eq 1 shows Newtonian viscous behavior, whereas when *n* > 1, the fluid exhibits shear-thickening properties. Table 2 lists the measured fitting parameters for all of the liquids used in this research.

**Table 2:** Viscosity Parameters for the Xanthan Solutions; *K* and *N* are the Fitting Parameters Described in Equation 1 and Plotted in Figure 1b

solutions	*K*	*n*
X400	0.1611	0.375
X800	0.3034	0.323
X1500	0.6565	0.269
X2500	1.2416	0.242
water	N/A	1
glycerol 50%	N/A	1
glycerol	N/A	1

Although Xanthan solutions are categorized as shear-thinning liquids at higher Xanthan concentrations, they exhibit viscoelastic behavior. Viscoelastic liquids have a yield stress, τ_0_, which must be exceeded to trigger the flow. If the externally applied stress is lower than the yield stress, the material will deform elastically (or flow en masse like a rigid body).

One of the most convenient methods of characterization of viscoelastic behavior is the oscillatory shear method (also called a frequency sweep test), which involves varying applied stress or shear rate over time. We further carried out the frequency sweep tests and obtained the viscoelastic properties of the liquids. Frequency sweep results are usually presented on a graph with the frequency (angular) plotted on the *y*-axis and the modulus of storage, *G*′, and viscous modulus, *G*″, on the *x*-axis plotted on a logarithmic scale. *G*′ is a measure of energy stored and recovered per cycle of deformation, i.e., the extent of elastic behavior, and *G*″ is a measure of energy dissipated per cycle, i.e., the extent of viscous behavior. A high frequency simulates fast motion on a short timescale, whereas a low frequency simulates slow motion on a longer timescale or at rest.^25^

Figure 1d shows the obtained modulus of storage (*G*′) and the viscous modulus (*G*″) as functions of frequency for the samples. It is apparent from the results shown in Figure 1d that for solutions with lower Xanthan concentrations (i.e., X400 and X800), the elastic properties of the liquid are dominant compared to the viscous properties at a higher frequency range. In contrast, at lower frequencies, the viscous properties of the liquid become dominant. However, for solutions with higher Xanthan concentrations (X1500 and X2500), the storage modulus (or elasticity) is higher than the viscous modulus at all frequency ranges, so the crossover point between the storage and loss modulus was not captured in our experiments. This means that these solutions (X1500 and X2500) behaved as a viscoelastic liquid in different flow circumstances. In light of the discussed results, we know that the rheology of Xanthan solutions has both viscous and elastic parts, which can be dominant in different situations depending on the Xanthan concentration and flow circumstances (i.e., shear rate).

**Figure 3:**
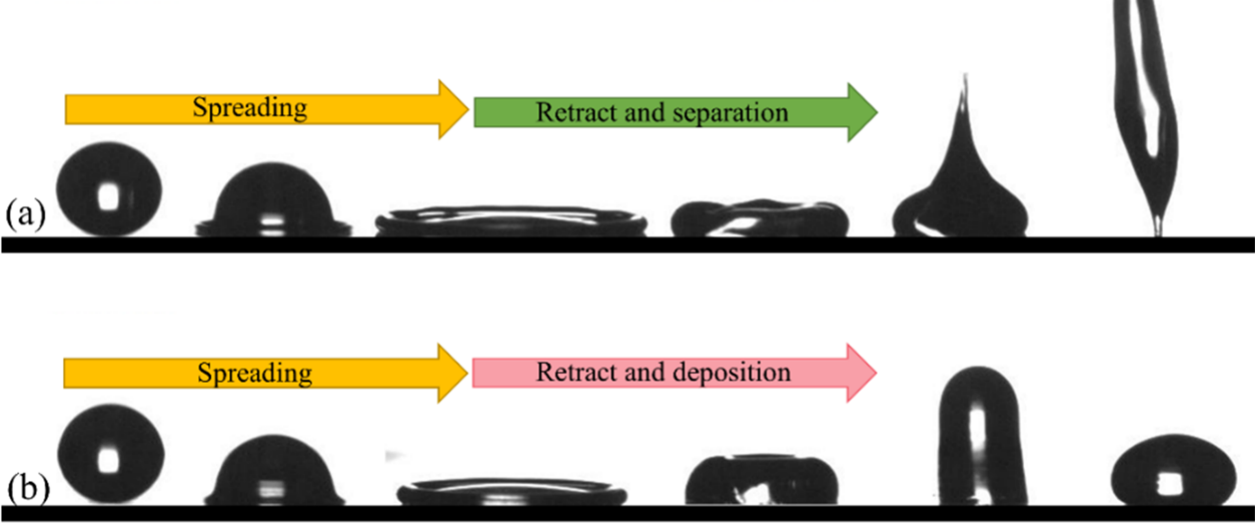
Snapshots of (a) 50% glycerol/water solution and (b) glycerol impact on the Glaco-coated surface. In all experiments, a droplet with a volume of 3.6 μL impacts the solid surface with a velocity of 1.4 m/s.

Water viscosity is two orders of magnitude lower than the apparent viscosity of the X1500 liquid. Besides, water does not exhibit viscoelastic behavior. When comparing the impact behaviors of X1500 and X2500 liquids to that of water droplets, it is not possible to definitively conclude that the observed differences in their impact dynamics are solely due to high viscous dissipation caused by the higher apparent viscosity of the solutions, nor can we confirm that the viscoelastic behavior is the sole cause of the differences. Hence, we repeated the same impact experiments with aqueous glycerol solutions to analyze the effect of viscous dissipation on the impact dynamics. As shown in Figure 1c, the viscosity of glycerol has the same order as the viscosity of Xanthan solutions for a wide range of shear rates. Therefore, it can be applied as a good reference to study the effect of apparent viscosity on the impact dynamics. In our experiments, we used 50% glycerol–water solutions and glycerol. Both glycerol and its water-based solutions (e.g., with glycerol concentration lower than 60%) exhibit a Newtonian behavior, which means that their viscosity remains constant, regardless of the shear rate or stress applied to them.^33^ When changing from water and Xanthan solutions to glycerol solutions while using the same experimental setup, the size of the generated droplets may vary. This variation is due to the fact that the droplet size in our experimental setup is primarily determined by the liquid density, surface tension, and needle size.^35^ In this phase of our research, our goal is to analyze the shape changes of the droplet during the impact. We are not trying to compare the impact parameters (such as contact time, maximum spreading, etc.) at this stage. However, to maintain consistency with the results from water and Xanthan/water solutions, we used a needle with a larger diameter when working with glycerol solutions to ensure that the droplet size remained (almost) the same. Our image processing analysis indicated that the droplet volumes for the 50% glycerol solution and pure glycerol were 3.4 and 3.2 μL, respectively. Figure 3a illustrates the snapshots of the impact of the glycerol/water solution on the superhydrophobic surface. This impact resulted in a liquid jet shape, similar to a water droplet. It should be noted that impact parameters such as the contact time and maximum spreading diameter are not much different from water. The impact outcome of the glycerol droplet, however, was categorically different. Since the viscosity of glycerol is two orders of magnitude higher than water and the water/glycerol solution (see Figure 1c), during the impact, more energy was dissipated, and the droplet did not contain enough energy to separate from the surface at the end of the retracting phase. The detailed analysis of the effect of viscosity on the impact dynamics of Newtonian liquids is not the key scope of this research. Therefore, we refer the readers to the recent work of Tai et al.^36^ for more information. Clearly, the snapshots shown in Figure 3 illustrate that an increase in the apparent viscosity of the liquid would not result in irregular droplet shapes at its separation stage. In light of these results, we now focus on the viscoelastic behavior of the liquid in the following section to explain the Xanthan solution droplet impact.

For all Xanthan solutions considered in Figure 2, we observed that the droplet shape becomes asymmetrical during the impact. Increasing the Xanthan concentration makes the droplet shape asymmetry at the separation points more evident. The asymmetric droplet impact shapes can be explained by considering the distribution of the Xanthan polymers inside the liquid. Practically it is difficult to achieve perfectly uniform Xanthan solutions.^37^ Even if these rod-like Xanthan polymer chains might be distributed uniformly in the liquid in the preparation stage, the liquid is under intense pressure during the droplet generation and impact process throughout the experiments. Thus, one can assume that the distribution of the polymer chains in the prepared droplet (before the free fall) is not uniform. These polymers behave like spring-like structures inside the liquid, which store energy under stress and simultaneously release the stored energy as the stress disappears. The nonuniform distribution of the polymers inside the liquid incites the generation of the local high-energy and low-energy regions inside the liquid, which breaks the symmetry of the internal recirculation field inside the droplet and triggers asymmetric rebounce of the droplet.

**Figure 4:**
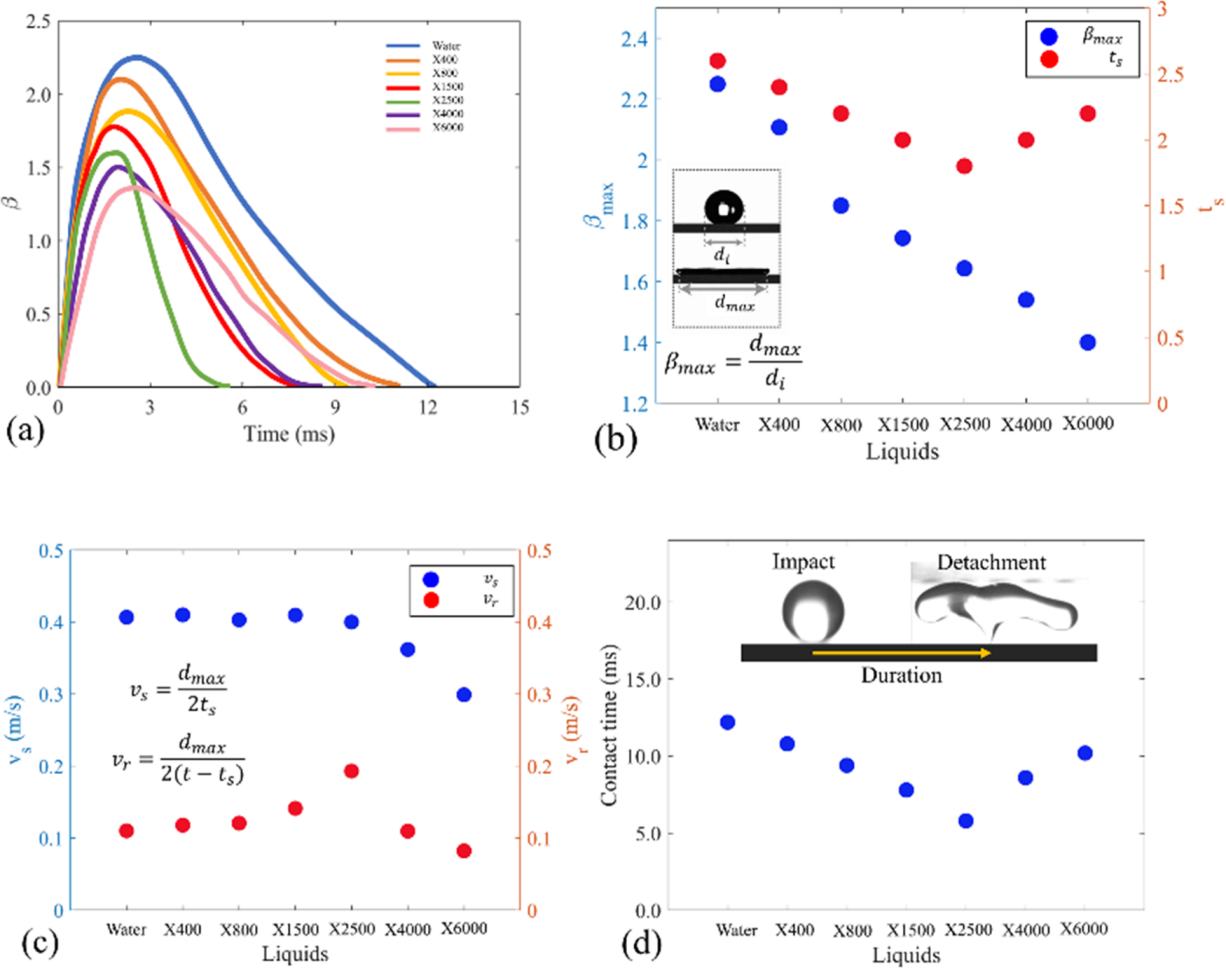
(a) Temporal evolution of contact width during the impact. (b) Maximum spreading ratio and spreading time as a function of Xanthan concentration in water. (c) Spreading and retracting velocities of the contact line for different liquids shown in panel (a). (d) Contact times for all of the liquids in the experiment. In all of the cases shown in this figure, a droplet with a volume of 3.6 μL is impacting the superhydrophobic surface with an impact velocity of 1.4 m/s.

Adding Xanthan to water does not only affect the apparent shapes of the droplet during the impact. The impact characteristic parameters such as the contact time and normalized maximum spreading diameter, β_max_ (β_max_ ≡ *d*_max_/*d*_i_, where *d*_max_ is the maximum spreading diameter and *d*_i_ is the initial droplet diameter before the impact), are also affected by the polymer chains. Figure 4a illustrates the temporal evolution of the contact line width, β (i.e., the width of the contact area with the solid surface normalized by the initial diameter of the droplet) dynamics during the impingement for a selected number of solutions. The contact line width increases until reaching a maximum value for all of the liquids during their spreading phase. This forms a thin film of liquid between the droplet and the surface. For all of the liquids, the maximum spreading radius (β_max_) is decreased by increasing the Xanthan concentration (see Figure 4b). There are two main reasons for this phenomenon. First, polymers inside the liquid create resistance against droplet spreading. As a result, increasing the concentration of Xanthan causes the maximum distance the droplet can spread to decrease. Second, when the apparent viscosity of the liquid is higher, more energy is dissipated during its spreading process, which limits the maximum spreading distance of the droplet. When the Xanthan concentration is increased up to 2500 ppm (as shown in Figure 4b), the spreading time, *t*_s_, decreases because the liquid spreading diameter becomes shorter. However, if the Xanthan concentration is increased beyond this point, the spreading time, *t*_s_, begins to increase. Although the distance that the liquid travels during spreading is lower than other liquids, the high apparent viscosity of the liquid causes a higher energy dissipation rate. As a result, less kinetic energy is available during the spreading phase, which causes the spreading to become slower.

After reaching the maximum spreading point, the droplet recurs, and the contact line moves inward toward the impact point. In Figure 4a, the slope of the lines is an indicator of the velocity of the contact line. Therefore, by observing the slope of the lines, we can gain insights into how fast the droplet is spreading and retracting on the surface. As shown in Figure 4c, the average spreading velocity (*v*_s_ ≡ *d*_max_/2*t*_s_) for X2500, X1500, X800, X400, and water is ∼0.4 m/s. However, when the concentration of Xanthan in a liquid is higher than 2500, the average velocity at which the liquid spreads decreases as the concentration of Xanthan increases. To explain this, one must focus on the balance between energy dissipation (due to the viscous forces) and energy storage in polymer bars (due to the elastic behavior). Our results show that by increasing the Xanthan concentration (up to 2500 ppm), the increase in energy dissipation is covered with higher energy storage in elastic polymer bars. When the concentration of Xanthan exceeds 2500 ppm, the balance is disrupted, and viscous dissipation takes over, resulting in a decrease in the average spreading velocity.

However, a further increase in Xanthan concentration leads to a rise in average retract velocity. For liquids with higher Xanthan concentrations (i.e., X4000 and X6000), viscous dissipation reduces the amount of the available energy during the retract, and the average retract velocity starts to reduce.

When the rim of the droplet reaches its maximum spreading diameter, it starts to retract toward the impact point. The average retract velocity (*v*_r_ ≡ *d*_max_/2(*t* – *t*_s_), where *t* is the contact time) is one of the most important parameters controlling contact time and impact outcome. This velocity is relatively constant for water, X400, and X800 liquids. It is apparent from Figure 4b that the retraction velocity of the droplets is increased by increasing the Xanthan concentration from 800 ppm up to 2500 ppm. This is due to the elastic behavior of the droplet. At higher Xanthan concentrations, the elastic behavior of the liquid becomes dominant (see Figure 1d). The liquid behaves like an elastic rubber which stores energy during the spreading. By the start of the retraction phase, the stored elastic energy is instantaneously released into the system, promoting a faster retraction, which leads to a shorter contact time. However, the retraction velocity decreases by increasing the Xanthan concentration for liquids with higher Xanthan concentrations (i.e., X4000 and X6000). As mentioned earlier, for these liquids, the viscous dissipation rate in these liquids is much higher than the elastic energy released into the system. Therefore, the droplet rims move toward the impact point slower.

**Figure 5:**
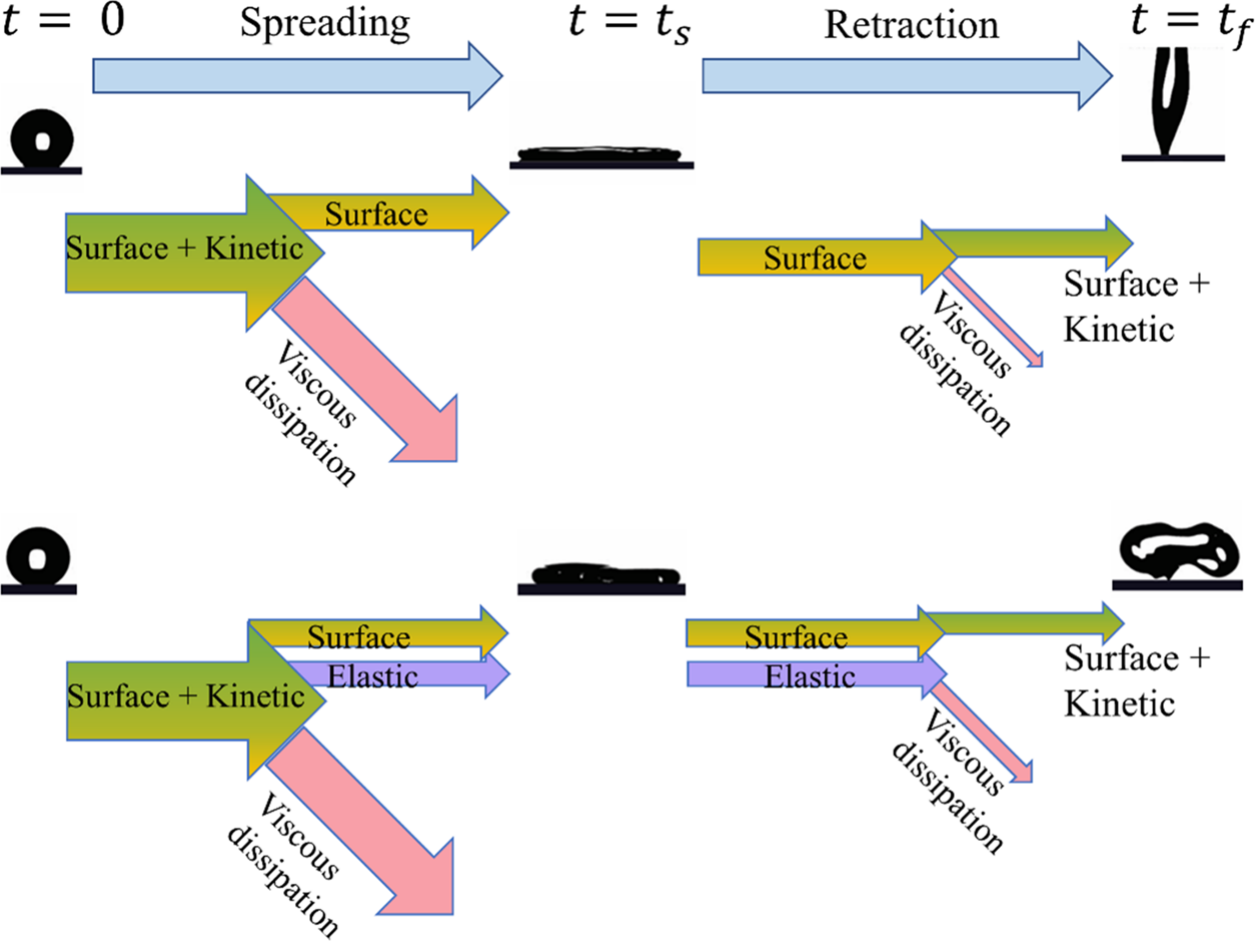
Schematic illustration of the energy budget during the impact for Newtonian and viscoelastic liquids.

Experimental studies by Tai et al. have shown that contact time increased when the apparent viscosity increased.^36^ However, Figure 4a illustrates that the contact time is reduced by adding Xanthan to water. For instance, the contact time of the X2500 droplet (which has an apparent viscosity two orders of magnitude higher than those of water at all shear rates) is ∼55% lower than water. This counterintuitive behavior can be explained by considering the changes in the rheology of the liquids. A higher concentration of Xanthan means that the apparent viscosity of the liquid is increased, and more energy is dissipated due to the internal recirculation (as a result of friction between liquid layers) and interaction between the liquid and solid surface (no-slip condition). At the onset of the impact, the liquid phase has an initial energy content that is the sum of surface energy (due to the existence of surface tension at the liquid–gas interface) and kinetic energy (due to the droplet inertia). Our previous numerical results have shown that ∼60 to ∼80% of the initial energy is dissipated during the spreading phase, and ∼10 to ∼20% of the energy is stored in the droplet in the form of surface energy.^14,38^ Once the retraction phase starts, the stored surface energy is converted into kinetic energy, which helps the droplet to retract and separate from the surface. A schematic illustration of energy conversions is illustrated in Figure 5. In viscoelastic liquids, however, the energy budget is different. Figure 1c shows that adding Xanthan increases the apparent viscosity of the liquid; thus, viscous dissipation during their spreading length is more significant for the Xanthan solutions. However, as explained, a part of the kinetic energy of the system is stored in the system as the elastic energy due to the elongation flows (i.e., forming flows that cause the fluid to stretch in one or more directions). By the start of the retraction, this elastic energy is instantaneously released into the liquid medium, which triggers the faster retraction of the contact lines and helps the droplet to separate from the surface in a shorter time. Although the Xanthan droplets are separated from the solid surface in a shorter time, they have less kinetic energy at the separation moment than water droplets. This is mainly due to the higher apparent viscosity of Xanthan solutions; therefore, the water droplet travels more in the vertical direction after the first rebounce (compared to Xanthan solutions).

**Figure 6:**
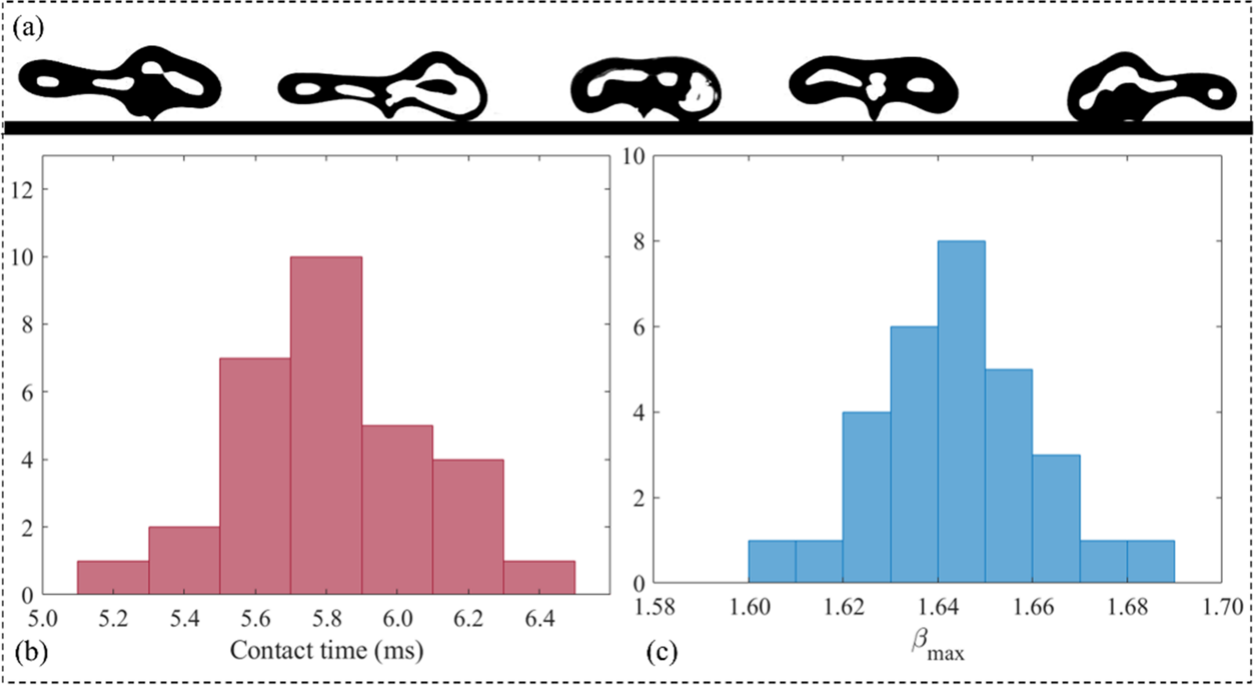
(a) Snapshots of the irregular shape of X2500 droplets at the separation moment. Frequency distribution of the (b) contact time and (c) maximum spreading diameter for an X2500 droplet impact. In all experiments, a droplet with a volume of 3.6 μL impacts the solid surface with a velocity of 1.4 m/s.

In our experiments, we observed that the shape of the Xanthan solution droplets became irregular throughout the impact, and these irregularities were clearly apparent at the separation moment. Figure 6a presents the shapes of the droplet at the separation moment. In light of the rheology analysis, we know that the nonuniform distribution of polymers inside the liquid causes this random shape of droplets. The next step in this research should be to distinguish the effect of this irregular impact on the contact time and β_max_. Therefore, we selected the X2500 liquid and carried out a statistical analysis of its impact processes. In our statistical analysis, we repeated the X2500 droplet impact on the superhydrophobic surface 30 times. In all of the impacts, the droplet volume and impact velocity were kept constant at 3.6 μL and 1.4 m/s, respectively. The results for the contact time and β_max_ are presented in Figure 6b,c. The average value of droplet contact time for 30 experiments is ∼5.81 ms with a standard deviation of ∼0.26 ms. Additionally, the mean value of β_max_ is ∼1.64 with a standard deviation of ∼0.017. Overall, the statistical analysis indicates that although the droplet shape varies slightly from experiment to experiment, the characteristic impact parameters remain relatively constant values.

### Effect of the *We* Number on Maximum Spreading

**Figure 7:**
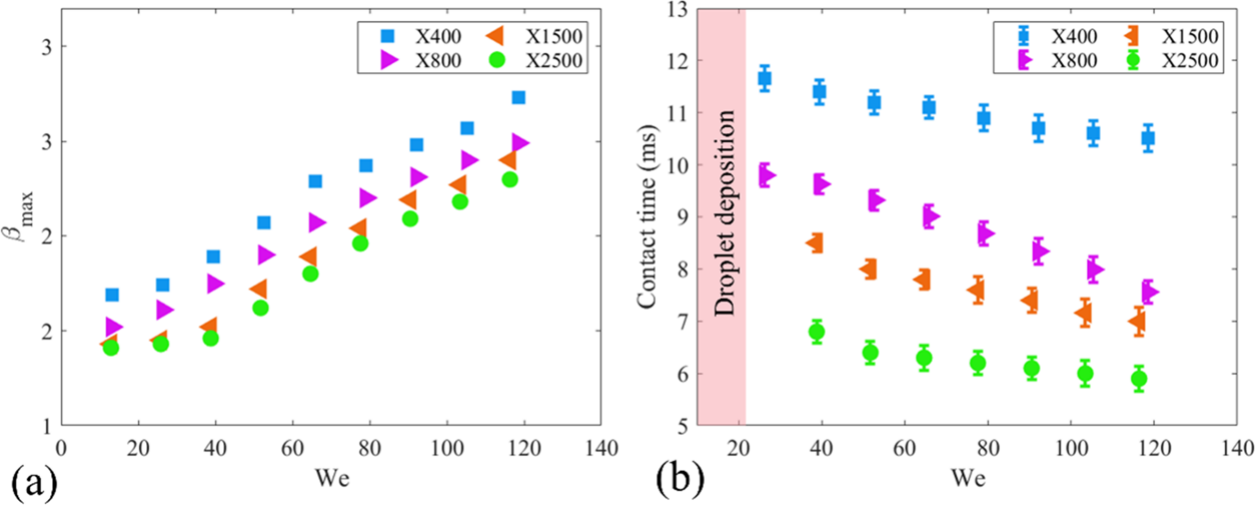
(a) Effect of the impact velocity on the droplet contact time. (b) Impact velocity vs the maximum spreading ratio. The definition of the maximum spreading ratio is illustrated in the embedded figure. The droplet volume is kept constant at 3.56 μL in all of the experiments.

We then focused on the effect of the impact velocity on the impact dynamics of non-Newtonian droplets. We carried out a set of systematic experiments using four types of non-Newtonian liquids (X400, X800, X1500, and X2500). In our experiments, we kept the droplet volume constant at 3.6 μL. By changing the droplet release height, we varied the impact velocity between 0.7 and 2.1 m/s. An important impact parameter that is critical for industrial applications is the maximum spreading ratio, β_max_. When viscous forces in a spreading droplet are negligible (in other words, when the viscosity of the droplet is comparatively low, for instance, water droplets), inertial forces and capillary forces are the only factors that limit the maximum spreading of droplets. Therefore, in these cases, maximum spreading is a key function of the Weber number (*We* = ρ*U*_0_^2^*D*_0_/σ, where ρ is the fluid density, *U*_0_ is the impact velocity, and σ is liquid surface tension).^39^ However, when the viscosity of the liquid is high, viscous forces play a major role in maximum spreading and should be considered. Recently, Tai et al.^40^ characterized the impact of Newtonian viscous liquids. They reported that increasing the *We* number increases the maximum spreading radius in liquids with higher viscosities. For the viscoelastic non-Newtonian liquids, however, the elastic energy plays a major role during the spreading, and the polymers inside the liquid are under stress during the spreading and store a part of the kinetic energy in the form of elastic energy. Therefore, viscous dissipation and elastic forces resist and limit the droplet spreading during the spreading. Therefore, the maximum spreading of liquid at the same *We* number (i.e., same impact velocity) is decreased by increasing Xanthan concentration. Besides, by increasing the impact velocity, since the droplet has higher kinetic energy at the onset of the impact, the maximum spreading is increased (see Figure 7a).

### Effect of the *We* Number on Contact Time

Figure 7b shows the effect of the *We* number on the droplet contact time. In this figure, the shaded areas show that droplets with low *We* numbers do not rebounce from the superhydrophobic surfaces. This is due to the low kinetic energy at the onset of the impact and high-energy dissipation during the spreading. The most surprising aspect of the data happens when the *We* number is higher than 24. Recent experimental results reported that the contact time was increased by increasing the impact velocities of the viscous droplets.^36^ In viscous Newtonian liquids, with increasing *We*, due to the higher initial kinetic energy of the droplet during the impact, internal recirculation is intensified inside the liquid, which leads to a sharp increase in viscous energy dissipation. As a result, the droplet contains lower energy at the end of the retraction phase, and the contact time is increased. However, as shown in Figure 7b, for the Xanthan solutions, the contact time is decreased by increasing the *We* number. To explain this counterintuitive behavior, it is important to understand that in liquids with shear-thinning rheology, increasing impact velocity leads to greater internal recirculation during spreading, which in turn increases the average shear rate during impact. As a result, the average apparent viscosity of the liquid during impact decreases (see Figure 1c). This decrease in viscosity leads to reduced energy dissipation, ultimately leading to a shorter contact time.

We further examined the effects of the impact velocity (and a high *We* number) on the droplet’s shape. Supporting Figure S3 displays the shapes of X400, X800, and X6000 liquids impacting onto a superhydrophobic surface with impact velocities of 2.43 and 2.8 m/s at the separation moment. Our findings demonstrate that increasing the impact velocity up to 2.8 m/s does not significantly alter the impact regime. Specifically, for X400, droplet separation occurs in an irregular shape. Besides, for the liquids with high concentrations of Xanthan (namely X1500, X2500, X4000, and X6000 liquids), regardless of the impact velocity, the resulting impact shape is a mushroom shape.

## Conclusions

In this study, we experimentally investigated the droplet impact of aqueous Xanthan solutions (non-Newtonian liquids) onto a superhydrophobic surface. We further investigated the effects of polymer concentration and impact velocity on the contact time and maximum spreading diameter. We observed that at higher Xanthan concentrations, the droplet was separated from the surface of the superhydrophobic surface with a mushroom cap-like shape. Our results showed that the contact time and maximum spreading diameter were significantly reduced at a higher concentration of Xanthan in water. We observed nonsymmetrical bouncing phenomena at the polymer solutions due to nonuniform polymer distribution inside the drop. Also, higher impact velocities resulted in shorter contact times and larger spreading diameters.

## Supporting Information Available

SEM images of the Glaco-coated surface before and after impacts; snapshots of X800, X4000, and X6000 solutions impacting on the Glaco-coated surface; and snapshots of X400 and X4000 droplets at the separation moment for scenarios with an initial impact velocity of 2.43 and 2.8 m/s (PDF)
