# Supplementary material for: Impact Dynamics of Non-Newtonian Droplets on Superhydrophobic Surfaces
Source: Langmuir. 2023 Apr 11;39(16):5793–802. doi: 10.1021/acs.langmuir.3c00043 (PMC10134492; doi:10.1021/acs.langmuir.3c00043)
Supplement: Supplementary file 1 — la3c00043_si_001.pdf [file la3c00043_si_001.pdf]

Impact dynamics of non-Newtonian droplet on superhydrophobic surfaces

Supplementary materials

Mehdi H. Biroun [1,2], Luke Haworth [2], Hossein Abdolnezhad [2], Arash Khosravi [3], Prashant Agrawal [2], Glen McHale,[4] Hamdi Torun [2], Ciro Semprebon [2], Masoud Jabbari [5], Yong-Qing Fu [2,*]

[1]Department of Chemical Engineering, University College London, London WC1E 7JE, UK.
[2]Faculty of Engineering and Environment, University of Northumbria, Newcastle upon Tyne NE1 8ST, UK.
[3]School of Mechanical Engineering, Iran University of Science and Technology, Iran.
[4] Institute for Multiscale Thermofluids, School of Engineering, University of Edinburgh, Kings Building, Edinburgh, EH9 3FB, UK.
[5] School of Mechanical Engineering, University of Leeds, Leeds LS2 9JT, UK.

*Corresponding author: Prof. Richard Y.Q. Fu, email: richard.fu@northumbria.ac.uk

Table of contents

Figure S1: SEM images of Glaco coated surface. a) Before impacts. b) After impacts S2

Figure S2: Snapshots of (a) X800, (b) X4000, and (c) X6000 solutions impacting on GLACO-coated surface. In all experiments, a droplet with a volume of 3.6 µl impacts the solid surface with a velocity of 1.4 m/s S2

Figure S3: Snapshots of X400 and X4000 droplets at the separation moment from the superhydrophobic surface for scenarios with an initial impact velocity of (a) 2.43 m/s, and (b) 2.8 m/s S3

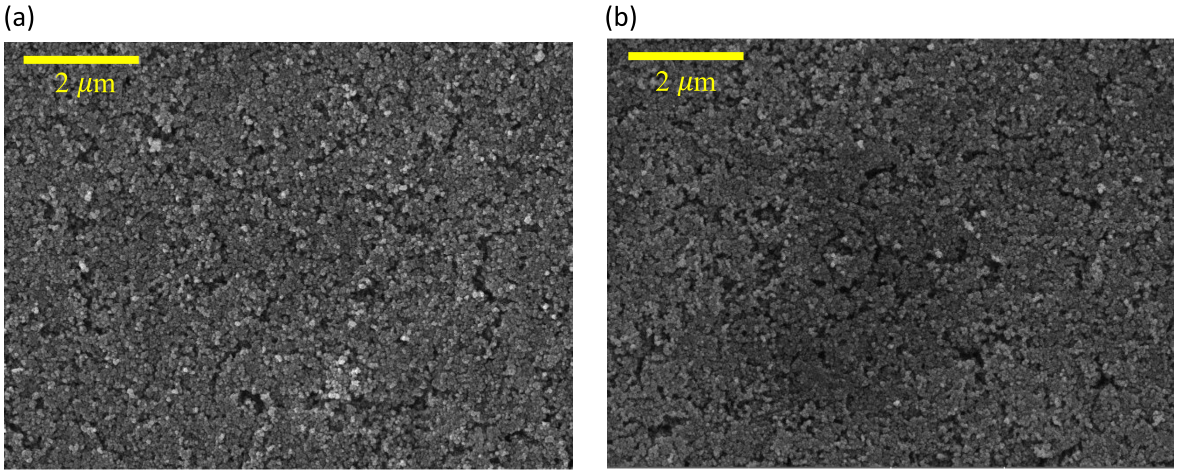

Figure S1: SEM images of Glaco coated surface. a) Before impacts. b) After impacts.

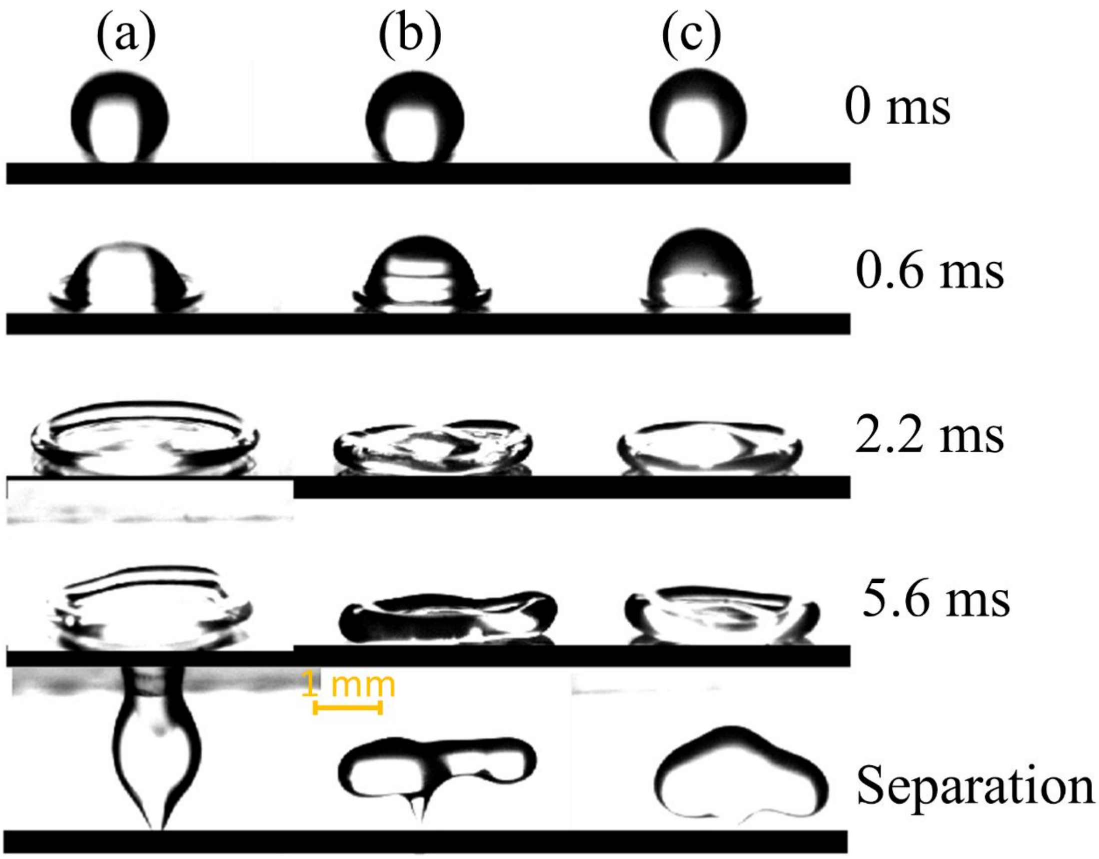

Figure S2: Snapshots of (a) X800, (b) X4000, and (c) X6000 solutions impacting on GLACO-coated surface. In all experiments, a droplet with a volume of 3.6 µl impacts the solid surface with a velocity of 1.4 m/s.

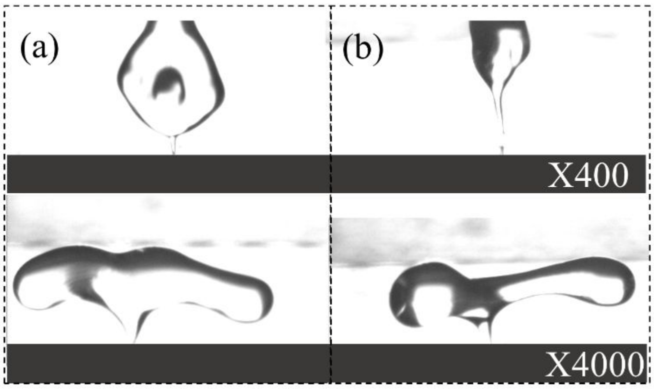

Figure S3: Snapshots of X400 and X4000 droplets at the separation moment from the superhydrophobic surface for scenarios with an initial impact velocity of (a) $2.43\ m/s$, and (b) $2.8\ m/s$.
